# Diurnal Rhythmicity Programs of Microbiota and Transcriptional Oscillation of Circadian Regulator, NFIL3

**DOI:** 10.3389/fimmu.2020.552188

**Published:** 2020-09-10

**Authors:** Masato Kubo

**Affiliations:** ^1^Division of Molecular Pathology, Research Institute for Biomedical Science, Tokyo University of Science, Noda, Japan; ^2^Laboratory for Cytokine Regulation, Center for Integrative Medical Science (IMS), RIKEN Yokohama Institute, Yokohama, Japan

**Keywords:** circadian rhythms, microbiota, metabolic diseases, cytokine, obesity

## Abstract

Circadian rhythms are a very exquisite mechanism to influence on transcriptional levels and physiological activities of various molecules that affect cell metabolic pathways. Long-term alteration of circadian rhythms increases the risk of cardiovascular diseases, hypertension, hypertriglyceridemia, and metabolic syndrome. A drastic change in dietary patterns can affect synchronizing the circadian clock within the metabolic system. Therefore, the interaction between the host and the bacterial community colonizing the mammalian gastrointestinal tract has a great impact on the circadian clock in diurnal programs. Here, we propose that the microbiota regulates body composition through the transcriptional oscillation of circadian regulators. The transcriptional regulator, NFIL3 (also called E4BP4) is a good example. Compositional change of the commensal bacteria influences the rhythmic expression of NFIL3 in the epithelium, which subsequently controls obesity and insulin resistance. Therefore, control of circadian regulators would be a promising therapeutic target for metabolic diseases.

## Introduction

Obesity is a major risk factor for several co-occurring diseases, including type II diabetes mellitus, non-alcoholic fatty liver disease, and ischemic cardiovascular disease, and the prevalence of these diseases has increased at an astounding rate in the past decades (1). About 44% of the global population is overweight, and more than 300 million individuals are affected by morbid obesity (2). This is thought to be the result of dramatic changes in the human lifestyle, ranging from a drastic change in dietary patterns, improved hygiene, and altered sleep cycles. Therefore, there is an urgent need to identify host and environmental factors that regulate human metabolism and energy homeostasis. In considering these two aspects, the intestinal flora is an environmental factor that greatly affects the body composition of mammals (3). The gut flora facilitates energy collection when energy derived from the diet is stored in adipose tissue (4). Recently, there is some evidence indicating a role for temporal and spatial dynamics in the community of microorganisms that inhabit the gastrointestinal tract. The circadian clock evolved in most species to adjust the physiology of the organism to daily environmental fluctuations (5–7). Epidemiological and experimental evidence has demonstrated that clock disturbances are linked to metabolic diseases, including obesity and hyperglycemia (8, 9). In understanding how regulation of microbial host metabolic pathways affects energy storage and body composition, we propose that the microbiota regulates body composition through the clock regulating transcription factor NFIL3 (also called E4BP4), which influences the circadian clock in intestinal epithelial cells through the regulation of group 3 innate lymphocyte cells (ILC3). This review describes how NFIL3 regulates body composition and establishes an essential network between the circadian clock and host metabolism.

## Circadian Clock and Metabolic Disorders

Circadian rhythms are a very exquisite mechanism by which organisms can adapt their behaviors to the 24-h light-dark (LD) cycle change in the external environment evoked by the rotation of the earth around the sun (10). Transcriptional levels and physiological activities of various molecules that affect cell metabolic pathways and organ functions have their own periodicity, which is known to be very consistent with the LD cycle of the external environment. Synchronizing the circadian clock with the metabolic system is necessary to make dietary substrates available for metabolic pathways that are energetically expensive and plays an important role in optimizing energy use. The intrinsic circadian clock is entrained by LD cycles, and the mammalian master clock resides in the suprachiasmatic nucleus (SCN), a small area of the anterior hypothalamus. The clock plays a role to drive oscillators distributed in various peripheral tissues through behavioral and neuroendocrine signals (11). Peripheral tissues have functional clock oscillators that are self-sustained and can operate independently of the central pacemaker and SCN rhythms (12). For instance, the temporal pattern of food intake influences the quality and quantity of the circadian transcriptome in the mouse liver. Therefore, while the LD cycle resets the master clock in the SCN, the timing of food intake can be a potent synchronizer of peripheral clocks.

Disruption of the circadian clock due to a genetic defect has been shown to have a strong causal relationship with metabolic diseases (13, 14). Indeed, sleep restriction or a 28-h LD cycle reduces insulin sensitivity and glucose tolerance (15). This has been demonstrated in epidemiological studies indicating that long-term alteration of sleep patterns increases the risk of obesity and metabolic disorders (16). Furthermore, the prevalence of cardiovascular diseases, hypertension, hypertriglyceridemia, and metabolic syndrome is higher in shift workers compared to day-time workers, and restriction of sleep time has been shown to increase the risk of obesity and diabetes. In this context, the temporal and spatial dynamics of the microbial flora may have a profound effect on host metabolism by tightly associating with the circadian clock.

A layer of intestinal epithelial cells (IECs) provides the physical barrier that separates commensal bacteria living in the intestinal lumen from the body. Interactions between commensal bacteria and Toll-like receptors (TLRs) members of the pattern recognition receptors (PRRs) are known to be mandatory for IEC homeostasis maintained by host-commensal symbiosis. Several functions in IECs, including nutrient absorption, cell proliferation, motility, and metabolic activities are regulated in a circadian rhythm. The circadian variation in the host intestine is tightly associated with diurnal oscillations of the intestinal microbiota colonizing the mammalian gastrointestinal tract, thus the oscillations of the circadian clock in IELs are controlled by the timing of food intake and the composition of the diet, which affects the expression pattern of some TLR genes. Moreover, the functional feature generated by the microbiota oscillations feeds into the circadian clock network not only in the intestine but also in the system as a whole (17).

Recent evidence indicates that alterations in the composition of the microbiome change the susceptibility to obesity of the host (18). Obesity and diabetes are known to cause chronic hyperglycemia in IELs, leading to a breakdown of barrier function that facilitated the alteration of intestinal bacterial components (19). Therefore, a chronic increase of glucose levels in obesity contributes to a detrimental effect in the maintenance of a barrier function in the IELs as a consequence of the functional reprogramming of metabolism and transcription. Subsequently, the perturbation in the coordinated daily interplay between the microbiota oscillations and the circadian transcriptional program in IELs enhances the risk for the development of obesity and systemic inflammation.

The composition and function of the bacterial community colonizing the mammalian gastrointestinal tract also undergo oscillations, which are affected by the dietary condition (14, 20, 21). The interaction between the host and gut microbiome can affect the circadian clock in different tissues (22, 23). Systemic metabolite rhythms and programming of transcriptional oscillations impact the homeostatic diurnal variation in the liver (22). These observations indicate that the microbiome is a major source of clock-modifying metabolites.

## Molecular Architecture of the Circadian Clock in the Gut

**FIGURE 1:**
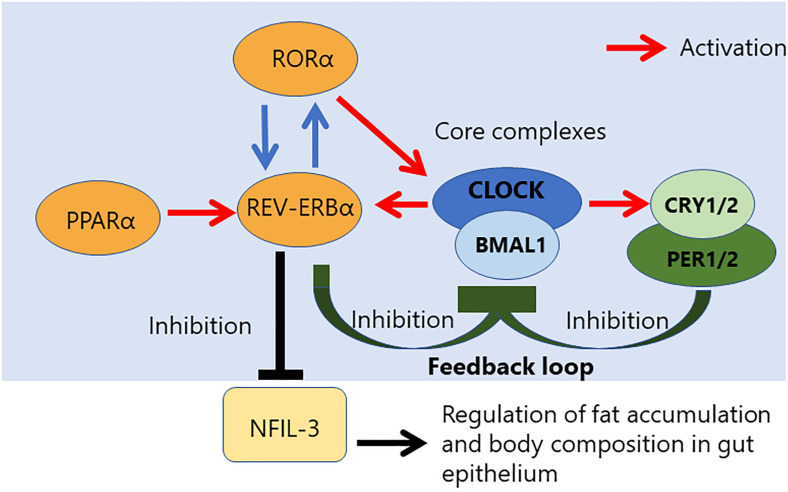
Relationship between NFIL-3 and the core complex controlling the circadian clock. Circadian rhythm is regulated by a transcriptional feedback loop composed of three core complexes. CLOCK/NPAS2/BMAL1 complex. NFIL-3 is a part of accessory loop, which regulates fat accumulation and body composition in gut epithelium.

Circadian rhythm is controlled by a core loop composed of the heterodimeric complex of two transcriptional activators, the circadian locomotor output cycles kaput (CLOCK), and brain and muscle ARNT-like 1 (BMAL1) (Figure 1) (24). The CLOCK and BMAL1 form the heterodimer via their HLH-PAS domains, and the heterodimer of transcriptional activators subsequently results in translocation to the nucleus, where it binds to an E-box sequence (CACGTG) in the promoter regions of repressors of CLOCK/BMAL1-mediated transcription, such as Period (PER 1 to 3) and Cryptochrome (CRY 1 and 2) genes. CRY inhibits the histone acetyltransferase p300, leading to a decrease of CLOCK/BMAL1-mediated transcription (25). The stability of the PER and CRY proteins is regulated by specific E3 ubiquitin ligase complexes, and the CLOCK/BMAL1 vs. CRY/PER mutually regulates each other via the central autoregulatory feedback loop (Figure 1). Therefore, this feedback loop is important to determine the periodicity of the circadian oscillation (26, 27).

Additional transcriptional feedback loops that are composed of members of the retinoic acid-related orphan nuclear receptor family, REV-ERBα/β repressor and RORα activator, ensure the stability and fidelity of the molecular clock (28). The antiphasic expression of the RORα and RevErbα controls a rhythmic expression of TLR in IELs (22). REV-ERB and its antagonist receptor RORα also competitively bind to the promoter region of the *Bmal1* gene to control rhythmic chromatin dynamics (29–32). The transcription regulator of lipid metabolism, peroxisome proliferator-activated receptor a (PPARα) is a critical activator of RevErbα expression (22). Activation of PPARα is known to promote many aspects of fatty acid metabolism. On the other hand, BMAL1 controls the rhythmic expression of short-chain fatty acids (SCFA) *receptor, Ffar2/3*, in the colonic muscle layer. Moreover, some evidence is indicating that diurnal microbial SCFA levels also influence on intestinal motility (33, 34).

The circadian rhythm associated genes directly contribute to the pathogenesis of intestinal diseases. Sleep disruption and chronic fatigue are the major complaints of patients with inflammatory bowel disease (IBD), and these symptoms affect the inflammatory process of the disease (35–37). The loss of BMAL1 disrupts both the circadian clock and the timing of regeneration in the mouse intestine controlled by TNF (38). Deficiency of Per1/2 results in not only decreased proliferation of intestinal stem cells (39) but also increased cell death of intestinal epithelial cells in the lower hemicrypts (40). In humans, a polymorphism in Per3 has been associated with increased susceptibility to and disease severity of IBD (41). Moreover, deficiency of Per1/2 controlled Wee1 plays a role in mitotic cell cycle arrest, resulting in increased susceptibility of the gut epithelium to inflammatory processes (42).

In the intestinal immune response, it has been reported that an essential role of gut-resident macrophages, particularly residents in lamina propria, contributes to host defense (43). The transcriptional profile in self-maintaining macrophage has a great impact on their localization in peripheral tissue. Therefore, impairment in diurnal rhythmicity programs of transcription may result in a reduction of intestinal functions.

## Role of the Clock Regulator NFIL3 and the Circadian Clock in the Immune System

NFIL3 was originally identified as a transcriptional repressor that binds to the E-box that controls the circadian clock (44); it is located in an auxiliary loop that exists outside the core loop (45). NFIL3 is a basic leucine zipper transcriptional factor that is mainly expressed in DC, T cells, and various other immune cells. This factor is required for the differentiation of the conventional dendritic cell 1 (cDC1) subset involved in cross-presentation (46, 47). Recently, NFIL3 expression was found to be required for the transition stage of cDC1 progenitors through the *Nfil3*–*Zeb2*–*Id2* pathway that controls the Irf8 enhancer switch (48). NFIL3 is expressed in common lymphoid progenitors (CLPs) and regulates the expression of Id2 and Eomes genes that are important for NK cell development (49, 50). Indeed, NFIL3 deficiency causes an intrinsic defect in NK cell development. NFIL3 also controls the differentiation of several other innate lymphoid cells (ILCs), including group 2 ILC (ILC2) and ILC3 cells, through the repression of Id2 in CLPs (51, 52). NFIL3 is a common regulator directing the development of CLPs that differentiate into all ILC lineages (53).

Regulation by circadian clocks has been described in the innate immune system because the CLOCK/BMAL1 complex regulates the expression of Toll-like receptor 9 (TLR9) and represses the expression of REV-ERBα, suppressing the induction of interleukin-6 (54). In contrast, the role of the circadian clock in the adaptive immune system seems to be controversial. Many previous studies indicated that the cell-intrinsic circadian clock is involved in different aspects of adaptive immune function. But, mice with a T-cell-specific deletion of Bmal1 had normal differentiation of T_*H*_17 cells (55), suggesting no intrinsic role of circadian clocks in the T cell response of the intestinal tract. On the other hand, NFIL3 was suggested to act as a repressor of a key driver of nuclear receptor RORgt, which is an essential intrinsic transcriptional factor for T_*H*_17 cell differentiation. Therefore, the diurnal expression of NFIL3 is regulated by the circadian network through direct repression of REV-ERBa, which binds to the consensus sequence of the *Nfil3* locus and represses NFIL3 expression (Figure 1). Therefore, NFIL3 expression in T cells plays a role in controlling the intestinal immune response regulated by T_*H*_17 cells (45). For T_*H*_1 cell immune function, NFIL3 has a different role to induce IL-10 and IL-13 expression (56) and, in this case, the expression of NFIL3 is induced by excessive IFN-γ stimulation to induce IL-10 and IL-13.

## Transcriptional Oscillation of NFIL3 in Intestinal Epithelial Cells

The defense at barrier surfaces by the gut epithelium plays a role in the containment of commensal bacteria. The barrier function of the gut epithelium is regulated by CD4^+^ T cell responses against commensal bacteria (57) and by antibacterial proteins derived from gut epithelial cells (58). NFIL3 is expressed by the small intestine epithelium under an LD cycle change, and this circadian expression is significantly altered in antibiotic-treated or germ-free mice (33). The expression of NFIL3 in the epithelium influences the response to commensal bacteria. Interestingly, intestinal epithelial cell-specific deficiency of NFIL3 has a great impact on the regulation of lipid storage and epididymal fat weight. Therefore, the expression of NFIL3 in the gut epithelium plays an important role in the regulation of lipid storage and body composition.

NFIL3 expression is known to be regulated by the core circadian clock transcriptional repressor REV-ERBα which binds to the *Nfil3* locus, resulting in circadian expression of NFIL3 (45, 59). Intestinal epithelial cells sense commensal bacteria via TLRs and the signaling adapter molecule, Myeloid differentiation primary response 88 (MyD88), and these innate signaling pathways promote NFIL3 expression via suppression of REV-ERBα expression in the epithelium. Interestingly, the expression pattern of REV-ERBα and NFIL3 in Zeitgeber time 4 (ZT4) are mutually exclusive in epithelial cells.

The DC-ILC3 network can be activated by flagellin or lipopolysaccharide (LPS) present in the outer membrane of Gram-negative bacteria (60, 61). The penetration of gram-negative bacteria into the intestinal epithelial barrier allows them to contact lamina propria DC and activates CD11c^+^ DC to produce interleukin 23 (IL-23). This process subsequently leads to further activation of group 3 innate lymphoid cells (ILC3s). ILC3s are reported to play an important role in defense at intestinal barrier surfaces via IL-17 and IL-22 production (62). The DC-derived IL-23 promotes IL-22 production by ILC3s, subsequently leading to the up-regulation of NFIL3 in the intestinal epithelium (63). In this case, NFIL3 plays a role to regulate lipid absorption and export in intestinal epithelial cells via promoting the expression of several molecules controlling lipid metabolism, including a member of the class B scavenger receptor family, CD36, which is a transporter of dietary fatty acids, stearoyl-coenzyme A-desaturase 1 (SCD1), a fatty acid hydroxylase, CYP2E1, and a fatty acid-binding protein 4 (FABP4) (64–67). This finding is consistent with the observation in a loss of function mutant of NFIL3 that lipid metabolism is partially altered (68).

Therefore, network regulation of the microbiota and the circadian clock in the intestinal tract is a critical process to control obesity and insulin resistance (18). Transcriptional oscillation of circadian regulators like NFIL3, which are controlled by the diurnal rhythmicity of the microbiota, is an important program for host metabolism.

## Author Contributions

The author confirms being the sole contributor of this work and has approved it for publication.

## Conflict of Interest

The author declares that the research was conducted in the absence of any commercial or financial relationships that could be construed as a potential conflict of interest.

## Abbreviations

BMAL1: brain and muscle ARNT-like 1
cDC1: conventional dendritic cell 1
CLOCK: circadian locomotor output cycles kaput
CLPs: common lymphoid progenitors
CRY: Cryptochrome
IBD: inflammatory bowel disease
ILCs: innate lymphoid cells
LD cycle: light-dark cycle
LPS: lipopolysaccharide
MYD88: Myeloid differentiation primary response 88
PER: period
ROR: retinoic acid receptor-related orphan receptor
SCN: suprachiasmatic nucleus
TLR9: toll-like receptor 9
ZT4: Zeitgeber time 4.

## References

[1] Després JP Lemieux I. Abdominal obesity and metabolic syndrome. *Nature.* (2006) 444:881–7. 10.1038/nature05488 17167477

[2] Ng M Fleming T Robinson M Thomson B Graetz N Margono C Global, regional, and national prevalence of overweight and obesity in children and adults during 1980-2013: a systematic analysis for the global burden of disease study 2013. *Lancet.* (2014) 384:766–81. 10.1016/S0140-6736(14)60460-8 24880830 PMC4624264

[3] Bäckhed F Ding H Wang T Hooper LV Koh GY Nagy A The gut microbiota as an environmental factor that regulates fat storage. *Proc Natl Acad Sci USA.* (2004) 101:15718–23. 10.1073/pnas.0407076101 15505215 PMC524219

[4] Turnbaugh PJ Ley RE Mahowald MA Magrini V Mardis ER Gordon JI. An obesity-associated gut microbiome with increased capacity for energy harvest. *Nature.* (2006) 444:1027–31. 10.1038/nature05414 17183312

[5] Hurd MW Ralph MR. The significance of circadian organization for longevity in the golden hamster. *J Biol Rhythms.* (1998) 13:430–6. 10.1177/074873098129000255 9783234

[6] Ouyang Y Andersson CR Kondo T Golden SS Johnson CH. Resonating circadian clocks enhance fitness in cyanobacteria. *Proc Natl Acad Sci USA.* (1998) 95:8660–4. 10.1073/pnas.95.15.8660 9671734 PMC21132

[7] Pittendrigh CS Minis DH. Circadian systems: longevity as a function of circadian resonance in Drosophila melanogaster. *Proc Natl Acad Sci USA.* (1972) 69:1537–9. 10.1073/pnas.69.6.1537 4624759 PMC426743

[8] Buxton OM Cain SW O’Connor SP Porter JH Duffy JF Wang W Adverse metabolic consequences in humans of prolonged sleep restriction combined with circadian disruption. *Sci Transl Med.* (2012) 4:129ra43. 10.1126/scitranslmed.3003200 22496545 PMC3678519

[9] Scheer FA Hilton MF Mantzoros CS Shea SA. Adverse metabolic and cardiovascular consequences of circadian misalignment. *Proc Natl Acad Sci USA.* (2009) 106:4453–8. 10.1073/pnas.0808180106 19255424 PMC2657421

[10] Reppert SM Weaver DR. Coordination of circadian timing in mammals. *Nature.* (2002) 418:935–41. 10.1038/nature00965 12198538

[11] Brown SA Azzi A. Peripheral circadian oscillators in mammals. *Handb Exp Pharmacol.* (2013) 217:45–66. 10.1007/978-3-642-25950-0_3 23604475

[12] Mendoza-Cózatl D Loza-Tavera H Hernández-Navarro A Moreno-Sánchez R. Sulfur assimilation and glutathione metabolism under cadmium stress in yeast, protists and plants. *FEMS Microbiol Rev.* (2005) 29:653–71. 10.1016/j.femsre.2004.09.004 16102596

[13] Thaiss CA Zeevi D Levy M Zilberman-Schapira G Suez J Tengeler AC Transkingdom control of microbiota diurnal oscillations promotes metabolic homeostasis. *Cell.* (2014) 159:514–29. 10.1016/j.cell.2014.09.048 25417104

[14] Leone V Gibbons SM Martinez K Hutchison AL Huang EY Cham CM Effects of diurnal variation of gut microbes and high-fat feeding on host circadian clock function and metabolism. *Cell Host Microbe.* (2015) 17:681–9. 10.1016/j.chom.2015.03.006 25891358 PMC4433408

[15] Van Cauter E Polonsky KS Scheen AJ. Roles of circadian rhythmicity and sleep in human glucose regulation. *Endocr Rev.* (1997) 18:716–38. 10.1210/edrv.18.5.0317 9331550

[16] Beccuti G Pannain S. Sleep and obesity. *Curr Opin Clin Nutr Metab Care.* (2011) 14:402–12. 10.1097/MCO.0b013e3283479109 21659802 PMC3632337

[17] Thaiss CA Levy M Korem T Dohnalová L Shapiro H Jaitin DA Microbiota diurnal rhythmicity programs host transcriptome oscillations. *Cell.* (2016) 167:1495–510. 10.1016/j.cell.2016.11.003 27912059

[18] Thaiss CA Itav S Rothschild D Meijer MT Levy M Moresi C Persistent microbiome alterations modulate the rate of post-dieting weight regain. *Nature.* (2016) 540:544–51. 10.1038/nature20796 27906159

[19] Thaiss CA. Microbiome dynamics in obesity. *Science.* (2018) 362:903–4. 10.1126/science.aav6870 30467161

[20] Liang X Bushman FD FitzGerald GA. Rhythmicity of the intestinal microbiota is regulated by gender and the host circadian clock. *Proc Natl Acad Sci USA.* (2015) 112:10479–84. 10.1073/pnas.1501305112 26240359 PMC4547234

[21] Zarrinpar A Chaix A Yooseph S Panda S. Diet and feeding pattern affect the diurnal dynamics of the gut microbiome. *Cell Metab.* (2014) 20:1006–17. 10.1016/j.cmet.2014.11.008 25470548 PMC4255146

[22] Mukherji A Kobiita A Ye T Chambon P. Homeostasis in intestinal epithelium is orchestrated by the circadian clock and microbiota cues transduced by TLRs. *Cell.* (2013) 153:812–27. 10.1016/j.cell.2013.04.020 23663780

[23] Murakami M Tognini P Liu Y Eckel-Mahan KL Baldi P Sassone-Corsi P. Gut microbiota directs PPARγ-driven reprogramming of the liver circadian clock by nutritional challenge. *EMBO Rep.* (2016) 17:1292–303. 10.15252/embr.201642463 27418314 PMC5007574

[24] Holtz Vitaterna M Turek FW. Chapter 12. Circadian clock genes. *Principles and Practice of Sleep Medicine* (Fifth Edition). ed. Kruger M. Roth T. Dement W. Amsterdam: Elsevier (2011). p. 141–50.

[25] Etchegaray JP Lee C Wade PA Reppert SM. Rhythmic histone acetylation underlies transcription in the mammalian circadian clock. *Nature.* (2003) 421:177–82. 10.1038/nature01314 12483227

[26] Duguay D Cermakian N. The crosstalk between physiology and circadian clock proteins. *Chronobiol Int.* (2009) 26:1479–513. 10.3109/07420520903497575 20030537

[27] Asher G Schibler U. Crosstalk between components of circadian and metabolic cycles in mammals. *Cell Metab.* (2011) 13:125–37. 10.1016/j.cmet.2011.01.006 21284980

[28] Guillaumond F Dardente H Giguère V Cermakian N. Differential control of Bmal1 circadian transcription by REV-ERB and ROR nuclear receptors. *J Biol Rhythms.* (2005) 20:391–403. 10.1177/0748730405277232 16267379

[29] Aguilar-Arnal L Hakim O Patel VR Baldi P Hager GL Sassone-Corsi P. Cycles in spatial and temporal chromosomal organization driven by the circadian clock. *Nat Struct Mol Biol.* (2013) 20:1206–13. 10.1038/nsmb.2667 24056944 PMC3885543

[30] Koike N Yoo SH Huang HC Kumar V Lee C Kim TK Transcriptional architecture and chromatin landscape of the core circadian clock in mammals. *Science.* (2012) 338:349–54. 10.1126/science.1226339 22936566 PMC3694775

[31] Perelis M Ramsey KM Marcheva B Bass J. Circadian transcription from beta cell function to diabetes pathophysiology. *J Biol Rhythms.* (2016) 31:323–36. 10.1177/0748730416656949 27440914 PMC8985168

[32] Vollmers C Schmitz RJ Nathanson J Yeo G Ecker JR Panda S. Circadian oscillations of protein-coding and regulatory RNAs in a highly dynamic mammalian liver epigenome. *Cell Metab.* (2012) 16:833–45. 10.1016/j.cmet.2012.11.004 23217262 PMC3541940

[33] Segers A Desmet L Thijs T Verbeke K Tack J Depoortere I. The circadian clock regulates the diurnal levels of microbial short-chain fatty acids and their rhythmic effects on colon contractility in mice. *Acta Physiol (Oxf).* (2019) 225:e13193. 10.1111/apha.13193 30269420

[34] Segers A Desmet L Sun S Verbeke K Tack J Depoortere I. Night-time feeding of Bmal1-/- mice restores SCFA rhythms and their effect on ghrelin. *J Endocrinol.* (2020) 245:155–64. 10.1530/joe-20-0011 32045364

[35] Ranjbaran Z Keefer L Farhadi A Stepanski E Sedghi S Keshavarzian A. Impact of sleep disturbances in inflammatory bowel disease. *J Gastroenterol Hepatol.* (2007) 22:1748–53. 10.1111/j.1440-1746.2006.04820.x 17914945

[36] Ananthakrishnan AN Long MD Martin CF Sandler RS Kappelman MD. Sleep disturbance and risk of active disease in patients with Crohn’s disease and ulcerative colitis. *Clin Gastroenterol Hepatol.* (2013) 11:965–71. 10.1016/j.cgh.2013.01.021 23376797 PMC3659204

[37] Ali T Madhoun MF Orr WC Rubin DT. Assessment of the relationship between quality of sleep and disease activity in inflammatory bowel disease patients. *Inflamm Bowel Dis.* (2013) 19:2440–3. 10.1097/MIB.0b013e3182a0ea54 23945186

[38] Stokes K Cooke A Chang H Weaver DR Breault DT Karpowicz P. The circadian clock gene BMAL1 coordinates intestinal regeneration. *Cell Mol Gastroenterol Hepatol.* (2017) 4:95–114. 10.1016/j.jcmgh.2017.03.011 28593182 PMC5453906

[39] Karpowicz P Zhang Y Hogenesch JB Emery P Perrimon N. The circadian clock gates the intestinal stem cell regenerative state. *Cell Rep.* (2013) 3:996–1004. 10.1016/j.celrep.2013.03.016 23583176 PMC3982394

[40] Gavrila AM Robinson B Hoy J Stewart J Bhargava A Amir S. Double-stranded RNA-mediated suppression of Period2 expression in the suprachiasmatic nucleus disrupts circadian locomotor activity in rats. *Neuroscience.* (2008) 154:409–14. 10.1016/j.neuroscience.2008.04.032 18511208

[41] Mazzoccoli G Palmieri O Corritore G Latiano T Bossa F Scimeca D Association study of a polymorphism in clock gene PERIOD3 and risk of inflammatory bowel disease. *Chronobiol Int.* (2012) 29:994–1003. 10.3109/07420528.2012.705935 22881285

[42] Pagel R Bär F Schröder T Sünderhauf A Künstner A Ibrahim SM Circadian rhythm disruption impairs tissue homeostasis and exacerbates chronic inflammation in the intestine. *FASEB J.* (2017) 31:4707–19. 10.1096/fj.201700141RR 28710114 PMC6159707

[43] Schepper SD Verheijden S Lizarraga JA Viola MF Boesmans W Stakenborg N Self-maintaining gut macrophages are essential for intestinal homeostasis. *Cell.* (2018) 175:400–15.e13. 10.1016/j.cell.2018.07.048 30173915

[44] Mitsui S Yamaguchi S Matsuo T Ishida Y Okamura H. Antagonistic role of E4BP4 and PAR proteins in the circadian oscillatory mechanism. *Genes Dev.* (2001) 15:995–1006. 10.1101/gad.873501 11316793 PMC312673

[45] Yu X Rollins D Ruhn KA Stubblefield JJ Green CB Kashiwada M TH17 cell differentiation is regulated by the circadian clock. *Science.* (2013) 342:727–30. 10.1126/science.1243884 24202171 PMC4165400

[46] Kashiwada M Pham NL Pewe LL Harty JT Rothman PB. NFIL3/E4BP4 is a key transcription factor for CD8ɑ^+^ dendritic cell development. *Blood.* (2011) 117:6193–7. 10.1182/blood-2010-07-295873 21474667 PMC3122942

[47] Seillet C Jackson JT Markey KA Brady HJ Hill GR Macdonald KP CD8α+ DCs can be induced in the absence of transcription factors Id2, Nfil3, and Batf3. *Blood.* (2013) 121:1574–83. 10.1182/blood-2012-07-445650 23297132

[48] Bagadia P Huang X Liu TT Durai V Grajales-Reyes GE Nitschké M An Nfil3-Zeb2-Id2 pathway imposes Irf8 enhancer switching during cDC1 development. *Nat Immunol.* (2019) 20:1174–85. 10.1038/s41590-019-0449-3 31406377 PMC6707889

[49] Male V Nisoli I Kostrzewski T Allan DS Carlyle JR Lord GM The transcription factor E4bp4/Nfil3 controls commitment to the NK lineage and directly regulates Eomes and Id2 expression. *J Exp Med.* (2014) 211:635–42. 10.1084/jem.20132398 24663216 PMC3978281

[50] Geiger TL Abt MC Gasteiger G Firth MA O’Connor MH Geary CD Nfil3 is crucial for development of innate lymphoid cells and host protection against intestinal pathogens. *J Exp Med.* (2014) 211:1723–31. 10.1084/jem.20140212 25113970 PMC4144732

[51] Seillet C Rankin LC Groom JR Mielke LA Tellier J Chopin M Nfil3 is required for the development of all innate lymphoid cell subsets. *J Exp Med.* (2014) 211:1733–40. 10.1084/jem.20140145 25092873 PMC4144736

[52] Xu W Domingues RG Fonseca-Pereira D Ferreira M Ribeiro H Lopez-Lastra S NFIL3 orchestrates the emergence of common helper innate lymphoid cell precursors. *Cell Rep.* (2015) 10:2043–54. 10.1016/j.celrep.2015.02.057 25801035

[53] Yu X Wang Y Deng M Li Y Ruhn KA Zhang CC The basic leucine zipper transcription factor NFIL3 directs the development of a common innate lymphoid cell precursor. *eLife.* (2014) 3:e04406. 10.7554/eLife.04406 25310240 PMC4356142

[54] Curtis AM Bellet MM Sassone-Corsi P O’Neill LA. Circadian clock proteins and immunity. *Immunity.* (2014) 40:178–86. 10.1016/j.immuni.2014.02.002 24560196

[55] Hemmers S Rudensky AY. The cell-intrinsic circadian clock is dispensable for lymphocyte differentiation and function. *Cell Rep.* (2015) 11:1339–49. 10.1016/j.celrep.2015.04.058 26004187 PMC4464971

[56] Motomura Y Kitamura H Hijikata A Matsunaga Y Matsumoto K Inoue H The transcription factor E4BP4 regulates the production of IL-10 and IL-13 in CD4+ T cells. *Nat Immunol.* (2011) 12:450–9. 10.1038/ni.2020 21460847 PMC3494493

[57] Hepworth MR Monticelli LA Fung TC Ziegler CG Grunberg S Sinha R Innate lymphoid cells regulate CD4+ T-cell responses to intestinal commensal bacteria. *Nature.* (2013) 498:113–7. 10.1038/nature12240 23698371 PMC3699860

[58] Gallo RL Hooper LV. Epithelial antimicrobial defence of the skin and intestine. *Nat Rev Immunol.* (2012) 12:503–16. 10.1038/nri3228 22728527 PMC3563335

[59] Duez H van der Veen JN Duhem C Pourcet B Touvier T Fontaine C Regulation of bile acid synthesis by the nuclear receptor Rev-erbalpha. *Gastroenterology.* (2008) 135:689–98. 10.1053/j.gastro.2008.05.035 18565334

[60] Pickard JM Maurice CF Kinnebrew MA Abt MC Schenten D Golovkina TV Rapid fucosylation of intestinal epithelium sustains host-commensal symbiosis in sickness. *Nature.* (2014) 514:638–41. 10.1038/nature13823 25274297 PMC4214913

[61] Lindemans CA Calafiore M Mertelsmann AM O’Connor MH Dudakov JA Jenq RR Interleukin-22 promotes intestinal-stem-cell-mediated epithelial regeneration. *Nature.* (2015) 528:560–4. 10.1038/nature16460 26649819 PMC4720437

[62] Sonnenberg GF Monticelli LA Alenghat T Fung TC Hutnick NA Kunisawa J Innate lymphoid cells promote anatomical containment of lymphoid-resident commensal bacteria. *Science.* (2012) 336:1321–5. 10.1126/science.1222551 22674331 PMC3659421

[63] Wang Y Kuang Z Yu X Ruhn KA Kubo M Hooper LV. The intestinal microbiota regulates body composition through NFIL3 and the circadian clock. *Science.* (2017) 357:912–6. 10.1126/science.aan0677 28860383 PMC5702268

[64] Coburn CT Knapp FF Jr. Febbraio M Beets AL Silverstein RL Abumrad NA. Defective uptake and utilization of long chain fatty acids in muscle and adipose tissues of CD36 knockout mice. *J Biol Chem.* (2000) 275:32523–9. 10.1074/jbc.M003826200 10913136

[65] Cohen P Miyazaki M Socci ND Hagge-Greenberg A Liedtke W Soukas AA Role for stearoyl-CoA desaturase-1 in leptin-mediated weight loss. *Science.* (2002) 297:240–3. 10.1126/science.1071527 12114623

[66] Zong H Armoni M Harel C Karnieli E Pessin JE. Cytochrome P-450 CYP2E1 knockout mice are protected against high-fat diet-induced obesity and insulin resistance. *Am J Physiol Endocrinol Metab.* (2012) 302:E532–9. 10.1152/ajpendo.00258.2011 22185839 PMC3311288

[67] Hotamisligil GS Johnson RS Distel RJ Ellis R Papaioannou VE Thaiss CA. Uncoupling of obesity from insulin resistance through a targeted mutation in aP2, the adipocyte fatty acid binding protein. *Science.* (1996) 274:1377–9. 10.1126/science.274.5291.1377 8910278

[68] Cai L Wang Z Ji A Meyer JM van der Westhuyzen DR. Scavenger receptor CD36 expression contributes to adipose tissue inflammation and cell death in diet-induced obesity. *PLoS One.* (2012) 7:e36785. 10.1371/journal.pone.0036785 22615812 PMC3353961

